# Prevalence of Aeroallergens in Allergic Rhinitis in a Tertiary Care Hospital

**DOI:** 10.31729/jnma.5218

**Published:** 2020-11-30

**Authors:** Monika Pokharel, Bikash Lal Shrestha, Dharmendra Karn, Ashish Dhakal, Abha Kiran K.C., Krishna Sundar Shrestha, Sushan Shakya

**Affiliations:** 1 Department of Otorhinolaryngology and Head and Neck Surgery, Kathmandu University School of Medical Sciences Dhulikhel Hospital, Dhulikhel, Kavre, Nepal; 2 Department of Dermatology, Kathmandu University School of Medical Sciences Dhulikhel Hospital, Dhulikhel, Kavre, Nepal

**Keywords:** *allergic rhinitis*, *prevalence*, *skin prick test*

## Abstract

**Introduction:**

The prevalence of allergic rhinitis has increased significantly globally over the last two decades. Detection of sensitizing aeroallergens plays a crucial role in the diagnosis and management of this troublesome disease. This study aims to investigate the spectrum of aeroallergens sensitization in patients with allergic rhinitis in a tertiary care hospital.

**Methods:**

A descriptive cross-sectional study conducted in the Department of Otorhinolaryngology of our hospital between January 2016 to December 2019. Ethical approval was taken from the Institutional Review Committee (No: 210/19). Patients diagnosed with allergic rhinitis were enrolled using the convenience sampling technique. Data entry and analysis was done using IBM Statistical Package for Social Sciences version 20.0.

**Results:**

Among 170 patients, altogether 103 (60.6%) patients yielded positive responses on the skin prick test. The most prevalent aeroallergens were Lepidoglyphus 86 (50.60%), Dermatophagoides pteronyssinus 85 (50%), Dermatophagoides farina 82 (48.20%), Thyrophagus 50 (29.40%), Blomia 46 (27.10%), Acarus 43 (25.30%), cat dander 26 (15.30%), dog dander 24 (14.10%), cow and buffalo dander 20 (11.8%), ragweed 20 (11.8%), grass pollen 18 (10.60%) and mugwort 17 (10%).

**Conclusions:**

This study highlights that the frequency of aeroallergens based on skin prick test in patients presenting to a tertiary care hospital which showed the dominance of house dust mites, dog and cat hair, pollen, and grasses. Reduced exposure and training of patients about protection against these agents will possibly help in controlling the severity of allergic rhinitis in this region.

## INTRODUCTION

Allergic Rhinitis (AR) is a chronic allergen-specific, IgE-mediated hypersensitivity disorder affecting the nasal lining characterized by nasal congestion, rhinorrhea, sneezing, nasal itchiness, and/or postnasal drip.^1^ Its global prevalence has risen significantly over the last two decades^2–7^ accounting from 37.90% to 50.60%.^5^

The direct and indirect effects including the cost of treatment, impaired quality of life, and presence of co-morbidities due to AR cause significant impact on the public health system.^8,9^ Detection of aeroallergens sensitization plays a crucial role in diagnosis and management of this disorder.^10,11^ Skin Prick test (SPT) is a reliable and well-tolerated method for the diagnosis and is routinely performed to identify allergens in a clinical setting.^10,12^ Many studies have shown that the spectrum of aeroallergens is significantly diverse in different countries^13^ and even in different parts of a country.^14^

Hence, the current study aims to illustrate the prevalence of common aeroallergens in the patients diagnosed with allergicrhinitis at the Kathmandu University School of Medical Sciences Dhulikhel Hospital.

## METHODS

This was a descriptive cross-sectional study conducted in the Department of Otorhinolaryngology and Head & Neck surgery at the Kathmandu University Dhulikhel Hospital between January 2016 to December 2019. The study was approved by the Kathmandu University School of Medical Sciences Institutional Review Committee (No: 210/19). Patients diagnosed as AR according to the AR and its Impact on Asthma (ARIA guidelines 2019) and residing in Kavre district were enrolled in our study. Patients with Chronic rhinosinusitis, nasal polyposis, benign or malignant tumors of the nose and paranasal sinuses, or other known cases of non-allergic rhinitis like occupational rhinitis were excluded. Patients with pregnancy and drug-induced rhinitis, wheezy bronchitis, and bronchiectasis were also excluded from the study. Similarly, patients with the parasitic infestation, patients under treatment with antihistamines, steroids, and antileukotreines within 7 days period, patients under 18 years of age, severe eczema, dermographism, and those with a history of previous life-threatening anaphylaxis were not included. Convenience sampling was done and the sample size was calculated using the formula,

$$\begin{array}{ll} \text{n} & \text{= }{\text{Z}}^{\text{2}}\times \text{p}\times \text{(1}-\text{p)}/{\text{e}}^{\text{2}}\\ & \text{= }1.96^{\text{2}}\times 0.5\times (1-0.5)/\text{0}{\text{.08}}^{\text{2}}\\ & \text{= }150 \end{array}$$

Where,

n = required sample size
Z = 1.96 at 95% Confidence Interval (CI)
p = prevalence,50% from previous studies
e = margin of error, 8%

Taking a 10% non-respondent rate, the sample size becomes 165. However, 170 patients were enrolled in the study. The medical records containing demographic data, presenting symptoms, symptoms of co-morbidities, general medical history, drug use, occupational and environmental exposure, family history of allergy, and smoking were collected.

SPT was performed according to European guidelines.^15^ SPT was performed after patients had stopped taking long-acting antihistamines for more than 1 week and short-acting antihistamines and sympathomimetic drugs for 5 days before the test. In the present study, allergens were selected based on the plant species existing in the Kavre district and other possible allergens. The aeroallergens included in the test battery were *Lepidoglyphus destructor* (storage mite), *Dermatophagoides pteronyssinus, Dermatophagoides farinae, Thyrophagus, Blomia, Acarus,* cat dander, dog dander, cow and buffalo dander, horse dander, rat dander, *Artemisia douglasiana* (ragweed), *Poaceae* (grass pollen), *Artemisia vulgaris* (mugwort), *Hordeum vuigare* (barley), *Fraxinus* (ash) and *Ficus religiosa* (bodhi) tree pollen, *Betula* (birch) pollen, *Piantago* (plantain), *Alternaria alternata, Corylus* (hazel), and *Aspergillus.*

A small amount of allergen extract was placed on the volar aspect of the forearm and introduced into the skin with a lancet. The lancet was penetrated at a low angle and its tip was lifted gently to raise the epidermis, without inducing any bleeding. To avoid false-positive results, the drops were placed at least 3 cm apart from each other. The test areas were numbered with a skin marker. A positive control (0.1% histamine in phosphate-buffered saline) and negative control (physiological saline) were also included in the test. A separate lancet was used for each test. The test solution was wiped off immediately after the SPT with the help of an absorbent paper towel on the skin prick area and carefully pressing it on the skin, without blending the different dilutions. The mean wheal diameter was read at 15 minutes. Wheel diameter of more than 3 mm was considered positive. A positive histamine reaction (≥3 mm) and a negative saline control reaction (<3 mm) was considered for the validity of SPT. Positive response to at least one of the allergens was accepted as the presence of sensitization.

Data entry and analysis was done using IBM Statistical Package for Social Sciences version 20.0.

## RESULTS

**Figure 1:**
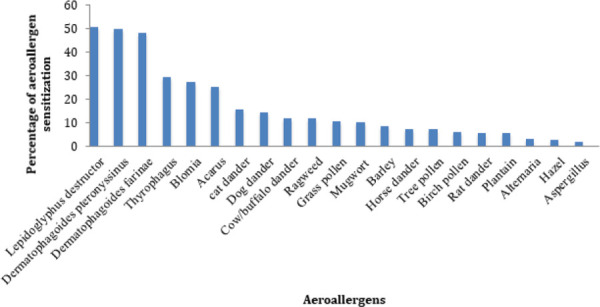
Prevalence of common aeroallergen sensitization in Kavre district.

Out of 170 enrolled participants, 103 (60.6%) patients yielded positive response on SPT. The most prevalent aeroallergens were *Lepidoglyphus* 86 (50.60%), *Dermatophagoides pteronyssinus* 85 (50%), *Dermatophagoides farina* 82 (48.20%), *Thyrophagus* 50 (29.40%), *Blomia* 46 (27.10%), *Acarus* 43 (25.30%), cat dander 26 (15.30%), dog dander 24 (14.10%), cow and buffalo dander 20 (11.8%), ragweed 20 (11.8%), grass pollen 18 (10.60%) and mugwort 17 (10%) (Figure 1).

There were 101 (59.40%) females and 69 (40.60%) males. The mean age was 31.84 years. The minimum age was 18 years, and the maximum was 66 years (Table 1).

**Table 1:** Sociodemographic characteristics of patients with AR (n = 170).

Variables	Category	Frequency n (%)
Sex	Male	69 (40.60)
	Female	101 (59.40)
Age (years)	18-20	20 (11.76)
	21-30	76 (44.70)
	31-40	41 (24.11)
	41-50	15 (8.82)
	51-60	12 (7.05)
	61-70	6 (3.52)
Family history of allergy	Present	68 (40)
	Absent	102 (60)
Smoking status	Present	36 (21.20)
	Absent	134 (78.80)
Pet ownership	Yes	40 (23.50)
	No	130 (76.50)

## DISCUSSION

In the current study, we revealed some interesting findings regarding the aeroallergens sensitization spectrum in patients with AR. We observed that Dermatophagoides pteronyssinus, *Dermatophagoides farinae, Lepidoglyphus destructor, Thyrophagus, Blomia, Acarus* which belong to a family of House Dust Mite (HDM)s were the predominant aeroallergens followed by dog and cat dander.

Our findings correspond with previously published studies in literature.^16,17^ This could be related to the high humidity and ambient temperatures which have been reported as optimal conditions for HDM propagation.^18^ Kavre district is located at a height of 1007 meters to 3018 meters from sea level and constitutes of 23.70%of the area belonging to the tropical region and 65.30% of the area constituting of the subtropical zone. The characteristics of the local climate are optimal for HDM propagation. Lifestyle may be another potential contributor to the high prevalence of sensitization to HDMs. Most of the Nepalese use cotton pillows, mattresses, and carpets which act as sources of HDM. At the same time, the exacerbating air pollution caused by automobile exhaust also contributes to the high detection of HDMs.^19^ Our study is in contrast with a study performed by Mohammadi et al.^20^ where the authors found that the most common allergens in that area were trees, weeds, grasses, and Dermatophagoides pteronysinus respectively. This could be explained by the fact that the spectrum of aeroallergens is significantly diverse in different countries and even in different parts of a country.^21^

Our study findings also illustrate the prevalence of plant pollen in Kavre. A possible explanation could be thatthere is a larger percentage of cultivated land in Kavre district. Hence, a greater proportion of people in this particular area are involved in farming for household or agricultural purposes, cutting grasses for feeding cattle and collecting firewood for cooking purposes which exposes them to plant-borne aeroallergens in particular pollen seasons.

Previous studies have shown that the more contacts one person has with pet allergens, the more possible he or she develops a symptom of airway hyper-reactivity.^22^ In our study, animal dander was also identified as an important risk factor for the development of AR. This finding is the following study by Wang J et al and in contrast with a study performed by Mohammadi et al.^20,23^ In Nepal, as in many other developing countries, many households keep cats and dogs as indoor pets. This could increase the chances of being exposed to dog and cat dander which act as allergens. This factor must be taken into account since avoiding contact with cat-dog dander allergens could probably help in the prevention of AR.

In the present study, the total positive rate of SPT was found to be significantly higher in women than in men. This finding may be due to our study population, which itself consisted of more women which are supported by a study in Turkey.^24^

This is in contrast with the study performed by Wang J et al ^23^ and Sattar HA et al.^25^ However, no convincing clarifications have been found in previous literature to suggest that sex leads to differential exposure to aeroallergens^26^ and no convincing clarifications have been given in previous literature regarding this aspect.

Our results demonstrated that the most frequently affected age group was between 20-40 with the highest peak in mid-twenties. Our findings are supported by a study by Boulet et al,^27^ where the authors suggested that the sensitization of all allergens tended to increase and reached its highest degree in young adults. Conversely, studies from other countries reported no difference in the prevalence of positive SPT in AR between younger and older patients.^28,29^

To the best of our knowledge, this is the first study addressing the prevalence of common aeroallergen sensitization patterns in patients with AR in the Kavre region. However, there are several limitations to this study. The study was conducted by retrospective analysis. This study was primarily a single-center study. Hence, we hope that more multicenter studies will be conducted in the future to reach a more accurate and comprehensive conclusion. We could not include some less common inhalant allergens in the SPT battery. Although in theory a patient may be solely sensitized to that particular aeroallergen not included in the test battery.

## CONCLUSIONS

This study demonstrated that the frequency of aeroallergens based on SPT in Kavre district showed the dominance of house dust mites, dog and cat hair, pollen, and grasses. Reduced exposure and training of patients about protection against these agents will possibly help in controlling the severity of AR in this region.

## References

[1] Bousquet J, Khaltaev N, Cruz AA, Denburg J, Fokkens WJ, Togias A (2008). Allergic Rhinitis and its Impact on Asthma (ARIA) 2008 update (in collaboration with the World Health Organization, GA(2)LEN and AllerGen). Allergy.

[2] Asher MI, Montefort S, Bjorksten B, Lai CK, Strachan DP, Weiland SK, Williams HIsaac Phase Three Study Group (2007). Worldwide time trends in the prevalence of symptoms of asthma, allergic rhinoconjunctivitis, and eczema in childhood: ISAAC Phases One and Three repeat multicountry cross-sectional surveys. Lancet.

[3] Pearce N, Ait-Khaled N, Beasley R, Mallol J, Keil U, Mitchell E, Robertson C (2007). Isaac Phase Three Study Group. Worldwide trends in the prevalence of asthma symptoms: phase III of the International Study of Asthma and Allergies in Childhood (ISAAC). Thorax.

[4] Bjorksten B, Clayton T, Ellwood P, Stewart A, Strachan D, Isaac Phase III Study Group. (2008). Worldwide time trends for symptoms of rhinitis and conjunctivitis: Phase III of the International Study of Asthma and Allergies in Childhood. Pediatr Allergy Immunol.

[5] Bunnag C, Jareoncharsri P, Tantilipikorn P, Vichyanond P, Pawankar R (2009). Epidemiology and current status of allergic rhinitis and asthma in Thailand -- ARIA Asia-Pacific Workshop report. Asian Pac J Allergy Immunol.

[6] Wong GW, Leung TF, Ko FW (2013). Changing prevalence of allergic diseases in the Asia-pacific region. Allergy Asthma Immunol Res.

[7] Silverberg JI (2017). Public health burden and epidemiology of atopic dermatitis. Dermatol Clin.

[8] Reed SD, Lee TA, McCrory DC (2004). The economic burden of allergic rhinitis: a critical evaluation of the literature. Pharmacoeconomics.

[9] Schoenwetter WF, Dupclay L, Appajosyula S, Botteman MF, Pashos CL (2004). Economic impact and quality-of-life burden of allergic rhinitis. Curr Med Res Opin.

[10] Seidman MD, Gurgel RK, Lin SY, Schwartz SR, Baroody FM, Bonner JR (2015). Clinical practice guideline: allergic rhinitis. Otolaryngol Head Neck Surg.

[11] Bousquet J, Bachert C, Canonica GW, Casale TB, Cruz AA, Lockey RJ (2009). Extended Global Allergy and Asthma European Network, World Allergy Organization and Allergic Rhinitis and its Impact on Asthma Study Group. Unmet needs in severe chronic upper airway disease (SCUAD). J Allergy Clin Immunol.

[12] Lee JE, Ahn JC, Han DH, Kim DY, Kim JW, Cho SH (2014). Variability of offending allergens of allergic rhinitis according to age: optimization of skin prick test allergens. Allergy Asthma Immunol Res.

[13] Bousquet PJ, Chinn S, Janson C, Kogevinas M, Burney P, Jarvis D (2007). Geographical variation in the prevalence of positive skin tests to environmental aeroallergens in the European Community Respiratory Health Survey I. Allergy.

[14] Arnedo-Pena A, García-Marcos L, García Herandez G, Aguinagua Ontoso I, Gonzalez Díaz C, Morales Suarez-Varela M (2005). Time trends and geographical variations in the prevalence of symptoms of allergic rhinitis in 6-7-year-old children from eight areas of Spain according to the ISAAC. An Pediatr (Barc).

[15] Bousquet J, Heinzerling L, Bachert C, Papadopoulos NG, Bousquet PJ, Burney PG (2012). Practical guide to skin prick tests in allergy to aeroallergens. Allergy.

[16] Li J, Sun B, Huang Y (2009). A multicentre study assessing the prevalence of sensitizations in patients with asthma and/or rhinitis in China. Allergy.

[17] Zhang C, Li J, Lai X, Zheng Y, Giesing B, Spangfort MD (2012). House dust mite and storage mite and storage mite IgE reactivity in allergic patients from Guangzhou, China. Asian Pac J Allergy Immunol.

[18] Andiappan AK, Puan KJ, Lee B, Nardin A, Poldinger M, Connolly J (2014). Allergic airway diseases in a tropical urban environment are driven by dominant mono-specific sensitization against house dust mites. Allergy.

[19] Wang W, Huang X, Chen Z (2016). Prevalence and trends of sensitisation to aeroallergens in patients with allergic rhinitis in Guangzhou, China: a 10-year retrospective study. BMJ Open.

[20] Mohammadi K, Gharagozlou M, Movahedi M (2008). A single center study of clinical and paraclinical aspects in Iranian patients with allergic rhinitis. Iran J Allergy Asthma Immunol.

[21] Oncham S, Udomsubpayakul U, Laisuan W (2018). Skin prick test reactivity to aeroallergens in adult allergy clinic in Thailand: a 12-year retrospective study. Asia Pac Allergy.

[22] Madhurantakam C, Nilsson OB, Uchtenhagen H, Konradsen J, Saarne T, Hogbom E (2010). Crystal structure of the dog lipocalin allergen Can f 2: implications for cross-reactivity to the cat allergen Fel D 4. J Mol Biol.

[23] Wang J, Zhou L, Chen Y, Luo R, Tao J (2012). Analysis of inhaled allergen spectrum of children with allergic rhinitis in Guangzhou. Lin Chung Er Bi Yan HouTou Jing Wai Ke Za Zhi.

[24] Ediger D, Günaydin FE, Erbay M, Şeker Ü (2020). Trends of sensitization to aeroallergens in patients with allergic rhinitis and asthma in the city of Bursa, South Marmara Sea Region of Turkey. Turk J Med Sci.

[25] Sattar HA, Mobayed H, al-Mohammed AA, Ibrahim AS, Jufairi AA, Balamurugan P, Mary VP, Bener A (2003). The pattern of indoor and outdoor respiratory allergens in asthmatic adult patients in a humid and desert newly developed country. Eur Ann Allergy ClinImmunol.

[26] Ezeamuzie CI, Thomson MS, Al-Ali S, Dowaisan A, Khan M (2000). Asthma in the desert: spectrum of the sensitizing aeroallergens. Allergy.

[27] Boulet LP, Turcotte H, Laprise C (1997). Comparative degree and type of sensitization to common indoor and outdoor allergens in subjects with allergic rhinitis and/or asthma. Clin Exp Allergy.

[28] Aydin S, Hardal U, Atli H (2009). An analysis of skin prick test reactions in allergic rhinitis patients in Istanbul, Turkey. Asian Pac J Allergy Immunol.

[29] Saleem N, Waqar S, Shafi A (2018). Skin Prick Test Reactivity to Common Aeroallergens among Allergic Rhinitis Patients. J Coll Physicians Surg Pak.

